# Report of the Integrative Molecular Cancer Epidemiology International Symposium, Lyon, France

**DOI:** 10.3332/ecancer.2008.95

**Published:** 2008-09-11

**Authors:** S Raimondi

**Affiliations:** Division of Epidemiology and Biostatistics, European Institute of Oncology, Via Ripamonti, 435, 20141Milan, Italy

## Abstract

An International Symposium on Integrative Molecular Cancer Epidemiology took place in Lyon, France, on 3–5 July 2008. The Symposium focused on aetiological and mechanistic aspects of molecular and genetic cancer epidemiology research and was divided into the following three sections:

1. Molecular epidemiology—application of novel molecular markers to cancer epidemiology.
2. Genomic epidemiology in the era of whole genome scan.
3. Integrative molecular epidemiology: visions for the future.

Participants included epidemiologists, geneticists, biochemical and molecular biologists, pharmacologists, pathologists and all researchers interested in this field. The Symposium provided a complete and clear overview of the present and future programmes in molecular cancer epidemiology. It also served to encourage international scientific collaboration between investigators working in this specific research field, and to stimulate transdisciplinary research with experts of other research areas. Highlights of each of the scientific presentations are summarized below.

## Introduction

Over the last two decades molecular epidemiology has developed as an independent discipline, which aims to overcome some limitations of traditional epidemiology and to offer a framework for applying novel molecular techniques to population and clinical studies. Furthermore, the development of the HAPMAP project and the advent of new technologies for genotyping hundreds of thousands of variants for a limited cost have led to a new generation of studies aimed at identifying genes associated with several diseases.

Cancer research has represented a good target area for the theoretical development and the application of molecular epidemiology due to the long duration of disease development, the heterogeneity of the relevant phenotypes, and the complexity of carcinogenic pathways.

An international symposium on Integrative Molecular Cancer Epidemiology took place in Lyon, France, on 3–5 July 2008, immediately before the 20th meeting of the European Association of Cancer Research. It provided an opportunity for investigators to present their work and to review the most promising areas of future development of molecular cancer epidemiology. The Symposium focused on aetiological and mechanistic aspects of molecular and genetic cancer epidemiology research, which have been addressed through the interplay of epidemiologists, clinicians and molecular biologists. Participants therefore included epidemiologists, geneticists, biochemical and molecular biologists, pharmacologists, pathologists and all the researchers interested in this field.

After the opening speech by Paolo Boffetta (IARC, Lyon), the first invited speaker, Frederica Perera, from Columbia University, New York, provided an overview of the main findings in molecular epidemiology from its first applications in cancer research in the early 1980s. A first example of a successful application was the measurement of chemical-specific DNA adducts formed by the interface between environmental exposures, like polycyclic aromatic hydrocarbons and DNA, a toxicological target. By measuring DNA adducts, researchers could identify individuals who are likely to be at increased risk for developing cancer after exposure to specific carcinogens. Further important findings included the association of chromosomal aberrations with cancer risk, and that individuals with specific genetic polymorphisms or nutritional deficits could be more susceptible to cancer. On the other hand, molecular epidemiology has failed to measure the full spectrum of pre-clinical alterations resulting from carcinogen exposure thus precluding clear gains in terms of cancer prevention. In order to address these gaps new epigenetic and ‘omic’ biomarkers have recently became available, but they need to be systematically validated using principles and criteria established over the past few decades in the epidemiology and molecular epidemiology of cancer. These new biomarkers can be used in combination with the earlier validated biomarkers of exposure, risk and susceptibility to identify ‘at risk’ individuals, increase our understanding of mechanistic carcinogenic pathways, and mount more effective intervention to prevent cancer occurrence.

In order to allow in-depth discussions of the different tasks, the Symposium was divided into the following three sections:

1. Molecular epidemiology—application of novel molecular markers to cancer epidemiology.
2. Genomic epidemiology in the era of whole genome scan.
3. Integrative molecular epidemiology: visions for the future.

## Molecular epidemiology—application of novel molecular markers to cancer epidemiology

Michael Pawlita (German Cancer Research Centre, DKFZ, Heidelberg) discussed newly developed high-throughput multiplex technologies for the simultaneous, quantitative detection of antibodies, up to 100 different viral or bacterial proteins, or the genomes of broad varieties of viruses and bacteria, providing some examples of their application to large cross-sectional, case-control and infection prevalence studies.

An overview on the application of epigenetics to cancer epidemiology was provided by Zdenko Herceg (IARC, Lyon), who highlighted that epigenetics play key roles in virtually all stages of cancer development and progression. The term ‘epigenetics’ refers to all heritable changes in gene expression and associated phenotypic traits that are not coded in the DNA sequence itself. Several critical processes found in cancer cells, such as silencing of tumour suppressor genes, activation of oncogenes, aberrant cell cycle, and defects in DNA repair, can be a consequence of not only genetic but also epigenetic changes, which could be induced by environmental, dietary and lifestyle factors. Epigenetic inheritance includes DNA methylation, histone modifications, and micro-RNAs. Epigenetic profiling using both genome-wide and candidate-gene approaches in different tumour types will help in elucidating the mechanism underlying tumourigenesis.

The next invited speaker, Anne-Lise Borresen-Dale (Institute for Cancer Research, Oslo) presented applications of expression micro-arrays on breast cancer epidemiology. By expression profiling on healthy and tumour breast tissue, they identified a gene signature of 82% accuracy, 87% sensitivity and 76% specificity for breast cancer diagnosis. They also found that the previously identified gene set efficiently discriminates breast cancer and non-breast cancer samples from a further study population, providing evidence for a gene expression signature as a potential additional tool in breast cancer diagnostic work-up.

Marco Pierotti (National Cancer Institute, Milan) focused his talk on gene expression analysis, by explaining how they tried to identify expression profiles potentially predictive of response to treatment, using an accessible source, such as blood. He used as an example the application of expression analysis to the study of toxicity from ionizing radiation therapy in cancer, with the hypothesis that some cases of toxicity could be associated with abnormal transcriptional response to radiation.

The last two presentations within the first session focused on proteomics. The first speaker, Samir Hanash (Fred Hutchinson Cancer Research Center, Seattle), discussed the current status of the field and emerging findings, underlying that current proteomic strategies allow quantitative profiling of cells, tissues and biological fluids, and identify proteins changes resulting from altered levels, post-translational modifications and amino acid substitutions. A major application of proteomics is assessment of health-related changes in the plasma proteome.

Roel Vermeulen (University of Utrecht) discussed the possibility of applying proteomics to epidemiological studies. The function of a cell can be described by the proteins that are present in the cell and the abundance of these proteins. Proteomics has theoretical advantages over genomics and transcriptomics; however, it might not be ready, at this point, to be used in large-scale (prospective) epidemiological research. This is due to major challenges that still need to be overcome both technologically (the ability to reliable detect a wide range of proteins) and epidemiologically (study design, sample collection, information on inter- and intra-individual variability).

Two proffered papers were further presented within the first section of the Symposium: the first one by KB Ribeiro (IARC, Lyon) was on serological response to HPV and the risk of head and neck cancer, while the second one by Q Wei (Anderson Cancer Center, Houston) presented the results from a study on *in vitro* benzo[a]pyren diol epoxide-induced damage to DNA and chromosomes as independent risk markers for squamous cell carcinoma of the head and neck.

## Genomic epidemiology in the era of whole genome scan

The second section was opened by Nazneen Rahman (The Institute of Cancer Research, Sutton, London) who presented her experience in breast cancer case-control mutation screening. She highlighted that there were three delineated components of the genetic architecture of breast cancer. The first ones are rare, high penetrance (>10-fold) autosomal dominant cancer predisposition genes such as BRCA1 and BRCA2; the second ones are common, low penetrance (<1.5-fold) susceptibility alleles that have emerged from genome-wide tag-SNP searches in breast cancer; and the third ones are rare, intermediate penetrance (2 to 4-fold) susceptibility alleles discovered through large-scale, case-control resequencing analyses. They have identified four DNA repair genes, ATM, CHEK2, BRIP1 and PALB2, which seem to play a role in breast cancer and exemplified this final class.

An application of Genome Wide Association studies (GWAS) to lung cancer was presented by Paul Brennan (IARC, Lyon). They conducted a GWAS on 317,139 SNPs in 1989 lung cancer cases and 2625 controls from six central European countries. They identified a locus in chromosome region 15q25 that was strongly associated with lung cancer. This locus was replicated in five separate lung cancer studies comprising an additional 2518 lung cancer cases and 4752 controls, and it was found to account for 14% of lung cancer cases. The association region contains several genes, including three that encode nicotinic acetylcholine receptor subunits (CHRNA5, CHRNA3 and CHRNB4).

The developed statistical approaches for GWAS were discussed by David Balding from Imperial College, London. He highlighted the limitations of most approaches that were simple one-SNP-at-time analysis with adjustments for population structure and with cryptic kins removed. He presented alternative approaches that could improve the power of this technique, by analysing multiple SNPs simultaneously with more sophisticated adjustments for population structure and cryptic kinship. Gene–gene and gene–environment interactions have not yet been widely reported but could be incorporated in future studies.

Xavier Estivill (CIBERESP and Pompeu Fabra University, Barcelona) highlighted the important role of copy number variants (CNV) in complex diseases like cancer. Several CNV are present in each individual and may contain genes with roles in response to the environmental and adaptation. It is therefore likely that regions containing CNVs might have important roles in drug-response, susceptibility to infection, inflammation and cancer. The mechanisms by which CNVs could have functional consequences include a direct gene dosage effect of the gene or genes embedded in the CNV, or a positional effect on genes proximal or distal to the CNV. By comparing samples from 12 population groups, they identified over 200 genomic regions that varied in the genomic structure between groups. Most of these regions coincided with already known CNVs and segmental duplications and contained genes with a role in immune response, adaptation to environment, and metabolic pathways.

Rodolfo Saracci (National Research Council, Pisa) discussed the applicability of molecular cancer epidemiology to public health. Until now, results from molecular cancer epidemiology studies have not been translated into significant prevention advances at the population level. However there is often a time-lag between novel research approaches and practical applications. Cervical cancer is the most relevant example of the recent practical application of molecular epidemiology: the aetiological link with human papilloma viruses has been established by advanced molecular epidemiology techniques, and the detection of the virus in cervical cells has come into discussion as complement to the pap-test for screening purposes. An ‘integrative epidemiology’ approach, which combines the study of environmental exposures, susceptibility biomarkers and biomarkers for molecular sub-typing of cancers, may lead to a more refined stratification of risk by individual traits and by cancer type, paving the way to individualized prevention.

Many interesting proffered papers and posters have been discussed within this session of the Symposium. Among them, two studies carried out within the European Prospective Investigation into Cancer and Nutrition cohort: the first one by A van Winden (The Netherlands Cancer Institute, Amsterdam) searched for early breast cancer biomarkers by serum protein profiling; the second one by M Jenab (IARC, Lyon) studied the circulating vitamin D concentration, Vitamin D Receptor polymorphisms and the risk of colorectal cancer. Other interesting papers include: one by HB Shen (Cancer Center of Nanjing Medical University) on genetic variants in fibroblast growth factor receptor 2 in breast cancer susceptibility in Chinese women; one by T. Hayashi (Radiation Effects Research Foundation, Hiroshima) on the effects of IL-10 and IL-6 gene polymorphisms and atomic-bomb radiation exposure on gastric cancer risk; one by E De Feo (Catholic University, Rome) presenting a case-control study on the effect of p53 and p73 polymorphisms on gastric cancer risk and progression in an Italian population and the special lecture by L Beretta on viral hepatitis and liver cancer.

## Integrative molecular epidemiology: visions for the future

The last session was opened by Nathaniel Rothman (National Cancer Institute, Bethesda), who discussed gene–environment interactions. He presented the results of a previously published study on the association between N-acetyl transferases 2 (***NAT2***) and gluthatione S-transferases-M1 (***GSTM1***) polymorphisms with bladder cancer risk. They evaluated heterogeneity among risk estimates by ethnic groups and assessed gene–smoking interaction. They found a significant interaction between ***NAT2*** and the number of cigarettes per day in bladder cancer risk by case-only analysis, while the risk estimate for ***GSTM1*** was not modified by ethnicity nor by smoking intensity.

Julia Ross (University of Minnesota Cancer Center, Minneapolis) presented a transdisciplinary study applied to epidemiology of childhood leukaemia. First, they investigated the potential role of environmental exposures (i.e. maternal folic acid, pesticides) in murine models through tracking disease outcome and gene methylation and expression in appropriate target cells in offspring. They identified 33 genes as differentially expressed between the offspring of mothers assigned to different dietary groups and selected five of these genes for validation by comparative RT-PCR. Second, they established a pregnancy cohort to investigate the functional relationship between specific exposures and their biomarkers in cord blood and neonatal blood spots in relation to relevant risk factors and genetic polymorphisms of interest.

John Ioannidis (University Ioannina School of Medicine, Ioannina) discussed how to evaluate cumulative evidence in genomic epidemiology. With the advent of massive-testing high-throughput platforms, there is an increasing amount and complexity of data; therefore, the concept of sufficient replication has evolved over time with more stringent criteria now required to claim that an association has a robust credibility. During a Venice meeting, criteria were proposed to appraise the evidence of genetic association; the criteria have three axes: amount of evidence, replication consistency and protection from bias. Each of the three axes is rated from A to C. Associations that get an A in all three axes are considered to have ‘strong’ epidemiological credibility; those that get at least one B but no C are considered to have ‘moderate’ epidemiological credibility and those that get at least one C are graded as having ‘weak’ credibility.

A transdisciplinary science approach for molecular epidemiology was discussed by Robert Hiatt (University of California, San Francisco). This approach is needed to integrate the study of the biological nature of cancer and its clinical applications with behavioural and social influences on cancer. Moreover, this may be a more effective way to apply the findings of molecular epidemiology for use in therapeutic, behavioural and public health interventions, by seeking to discover interactions between social, environmental, behavioural and biologic factors in cancer aetiology.

Gilles Thomas (National Cancer Institute, Bethesda) discussed the impact of whole genome scans on cancer research, which is especially important for less common cancers (i.e. pancreas and kidney cancer) and for sub-types predisposition (i.e. in colorectal and breast cancer). GWAS are useful to identify the locus of interest, but then it is necessary to search for the specific variant in that region, which is associated with the disease. Although this search could be done through linkage disequilibrium analysis, this could be hard to do for most regions for which there is not a candidate gene. For the future, it is necessary to develop new statistical methods for multi-SNPs association, build networks that explain and summarize the gene–environment interaction in different tumours, identify a reliable genetic risk model, which incorporates genotypes from multiple loci, and finally test a first-proposed simple model within international collaborative studies.

The congress was closed with Martyn Smith’s presentation (University of California, Berkeley) on the future perspectives of molecular cancer epidemiology. In the future, we will probably witness the final development of the ‘omic’ technologies, such as transcriptomics, proteomics and metabolomics. Also, the epigenome has not been fully explored to date, but new micro-arrays that measure hundreds of micro-RNAs or the CpG promoter methylation status of hundred of genes are now available. Finally, advances in nanotechnology should allow for multiplexed immuno-assay of protein adducts, cytokines and antibodies in very small quantities of human material. Since about 90% of cancer risk is explained by environmental factors and 10% by genetics, probably in the not-too-distant future molecular cancer epidemiology will be revolutionized by the emerging field of exposure biology.

## Conclusions

The Symposium was very useful in providing a complete and clear overview of the present and future of molecular cancer epidemiology because of very interesting presentations by all the speakers. It was also useful both to encourage international scientific collaboration between investigators working in this specific research field, and to stimulate transdisciplinary research with experts of other research areas.

